# 1,9-Bis(2-pyridyl)-1,2,8,9-tetrathia-5-oxanonane

**DOI:** 10.3390/M642

**Published:** 2009

**Authors:** Jeet Kalia, Ronald T. Raines

**Affiliations:** 1 Department of Biochemistry, University of Wisconsin–Madison, 433 Babcock Drive, Madison, WI 53706-1544, USA; 2 Department of Chemistry, University of Wisconsin–Madison, 1101 University Avenue, Madison, WI 53706-1322, USA

**Keywords:** crosslinking, cysteine, disulfide, protein

## Abstract

Disulfide crosslinking of proteins is typically performed by treating proteins bearing cysteine residues with small-molecule disulfide reagents. The process results in the formation of a mixed disulfide intermediate, which then reacts with the cysteine residue of another protein molecule to form the crosslinked product. This second step requires the intimate association of two large reactants. The ensuing steric hindrance can result in poor crosslinking yields. Here, we introduce a bis(disulfide) reagent in which activated disulfides are separated by linkers that can alleviate steric hindrance and thereby potentially increase the efficiency of crosslinking.

Disulfide bonds are the most prevalent natural covalent crosslinks in proteins. Formed between cysteine side chains, these linkages are crucial for the structural and functional integrity of proteins. For example, the four disulfides of ribonuclease A remain intact at extremely low pH and high temperatures, thereby contributing enormously to the legendary stability of this protein [1,2]. Disulfides also play a major role in the remarkable stability of thermophilic proteins [3]. In addition to their role as structural scaffolds, disulfides often serve as linkages between protein monomers that exist physiologically as oligomers [4].

Following Nature's lead, biochemists and chemical biologists have inserted disulfide bonds in proteins and peptides to increase their stability, and to link them together to form multimers [5,6]. Disulfide crosslinking has also been used as a tool to elucidate the structure of proteins [7], define the conformational changes that underlie protein function [8], and modulate enzymatic activity [9,10].

Most disulfide crosslinking experiments are performed by a two-step procedure. In the first step, a cysteine-bearing protein is treated with a labile disulfide reagent, such as 2,2′-dithiopyridine disulfide [11] or 5,5′-dithiobis(2-nitrobenzoic acid) [12]. This reaction results in the formation of a labile mixed disulfide intermediate. In the second step, this intermediate reacts with another protein molecule bearing a reactive cysteine to form the desired crosslinked product. This second step can be problematic because it entails a reaction between two large reactants that can be impeded by steric hindrance, compromising crosslinking yields.

To mitigate steric hindrance, we designed 1,9-bis(2-pyridyl)-1,2,8,9-tetrathia-5-oxanonane (**1**), a reagent that contains two labile disulfides separated by a linker. Upon treatment with a protein bearing a reactive cysteine, one disulfide of the reagent reacts with the protein to form a stable mixed disulfide. In a second step, the remaining labile disulfide reacts with a cysteine residue of another protein to form the desired crosslinked product. This method is likely to increase crosslinking efficiency compared to the conventional method.

The synthesis of bis(disulfide) **1** was accomplished in a single step with commercial starting materials (Scheme 1). We used the same route to avail a longer congener, 1,12-bis(2-pyridyl)-1,2,11,12-tetrathia-5,8-dioxadodecane (**2**). We chose oligoethylene oxides to serve as the linker within both reagents because oligo- and polyethylene oxides have favorable properties such as high water solubility, chemical inertness, and low toxicity [13]. Molecules containing polyethylene oxide backbones with various chain lengths are available. Hence, this strategy can be employed to generate reagents possessing a wide range of linker lengths, enabling utility in a variety of applications.

## Synthesis

### Materials

2,2′-Dithiopyridine disulfide, 3-oxa-1,5-disulfanylpentane, and 3,6-dioxa-1,8-disulfanyloctane were from Sigma–Aldrich (St. Louis, MO). Synthetic reactions were monitored by thin-layer chromatography with visualization by UV-light or staining with phosphomolybdic acid. Flash chromatography was performed with columns of silica gel 60, 230–400 mesh (Silicycle, Québec City, Québec, Canada).

### Instrumentation

NMR spectra were acquired with a Bruker AC+ 300 spectrometer (^1^H NMR: 300 MHz, ^13^C NMR: 75 MHz) at the Magnetic Resonance Facility in the Department of Chemistry at Madison. ^13^C spectra were proton-decoupled. Mass spectra were obtained with electrospray ionization (ESI) techniques.

### Synthesis of 1,9-bis(2-pyridyl)-1,2,8,9-tetrathia-5-oxanonane (**1**)

2,2′-Dithiopyridine disulfide (1.00 g, 4.44 mmol) was dissolved in 20 mL of dichloromethane, and to this solution was added 3-oxa-1,5-disulfanylpentane (0.32 g, 2.22 mmol). The color of the reaction mixture changed to yellow within 2 min after addition of the dithiol. The reaction mixture was stirred at room temperature for 6 h. Solvent was then removed by using a rotary evaporator at water aspirator pressure (<20 torr), and the residue was purified by flash chromatography (80% v/v hexane in ethyl acetate). Bis(disulfide) **1** was obtained as a translucent, yellow liquid in an unoptimized yield of 53%. ^1^H NMR (300 MHz, CDCl_3_) δ 8.48–8.44 (m, 2H), 7.78–7.73 (m, 2H), 7.68–7.61 (m, 2H), 7.11–7.05 (m, 2H), 3.67 (t, *J* = 6.3 Hz, 4H), 2.97 (t, *J* = 6.3 Hz, 4H) ppm; ^13^C NMR (75 MHz, CDCl_3_) δ 160.5, 149.5, 137.2, 120.8, 119.9, 68.9, 38.7 ppm. MS (ESI) *m*/*z* 357.2 (MH^+^ [C_14_H_17_N_2_OS_4_] = 357.55).

### Synthesis of 1,12-bis(2-pyridyl)-1,2,11,12-tetrathia-5,8-dioxadodecane (**2**)

2,2′-Dithiopyridine disulfide (0.94 g, 4.18 mmol) was dissolved in 20 mL of dichloromethane, and to this solution was added 3,6-dioxa-1,8-disulfanyloctane (0.40 g, 2.09 mmol). The color of the reaction mixture changed to yellow instantaneously upon addition of the dithiol. The reaction mixture was stirred at room temperature for 6 h. Solvent was then removed by using a rotary evaporator at water aspirator pressure (<20 torr), and the residue was purified by flash chromatography (70% v/v hexane in ethyl acetate). Bis(disulfide) **2** was obtained as a translucent, yellow liquid in an unoptimized yield of 33%. ^1^H NMR (300 MHz, CDCl_3_) δ 8.47–8.43 (m, 2H), 7.79–7.75 (m, 2H), 7.68–7.62 (m, 2H), 7.11–7.05 (m, 2H), 3.73 (t, *J* = 6.5 Hz, 4H), 3.56 (m, 4 H), 2.99 (t, *J* = 6.4 Hz, 4H) ppm; ^13^C NMR (75 MHz, CDCl_3_) δ 160.6, 149.7, 137.2, 120.8, 119.8, 70.5, 69.3, 38.6 ppm; MS (ESI) *m/z* 401.2 (MH^+^ [C_16_H_21_N_2_O_2_S_4_] = 401.6).

## Supplementary Material

Supporting_Information

## Figures and Tables

**Scheme 1:**
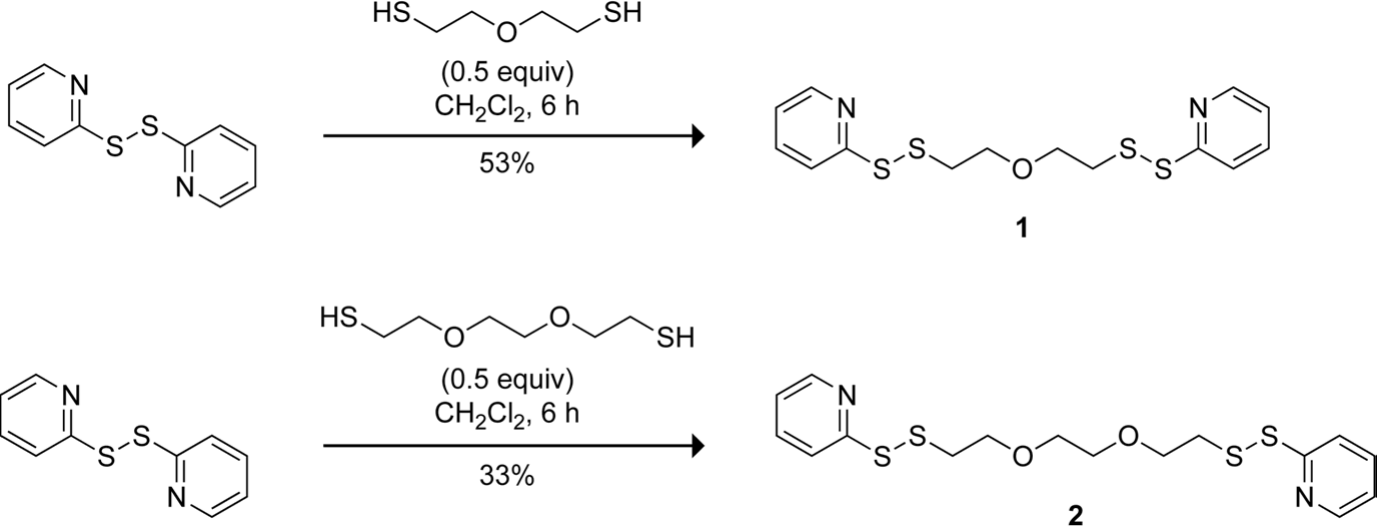
Route for the syntheses of bis(disulfide) **1** and **2**.

## References

[1] Klink TA, Woycechowsky KJ, Taylor KM, Raines RT (2000). Contribution of disulfide bonds to the conformational stability and catalytic activity of ribonuclease A. Eur. J. Biochem.

[2] Pecher P, Arnold U (2009). The effect of additional disulfide bonds on the stability and folding of ribonuclease A. Biophys. Chem.

[3] Beeby M, O'Connor BD, Ryttersgaard C, Boutz DR, Perry LJ, Yeates TO (2005). The genomics of disulfide bonding and protein stabilization in thermophiles. PLoS Biol.

[4] Kim J-S, Raines RT (1995). Dibromobimane as a fluorescent crosslinking reagent. Anal. Biochem.

[5] Kotch FW, Raines RT (2006). Self-assembly of synthetic collagen triple helices. Proc. Natl. Acad. Sci. USA.

[6] Ottl J, Moroder L (1999). Disulfide-bridged heterotrimeric collagen peptides containing the collagenase cleavage site of collagen type I. Synthesis and conformational properties. J. Am. Chem. Soc.

[7] Pakula AA, Simon MI (1992). Determination of transmembrane protein structure by disulfide crosslinking: The *Escherichia coli* Tar receptor. Proc. Natl. Acad. Sci. USA.

[8] Armstrong N, Jasti J, Beich-Frandsen M, Gouaux E (2006). Measurement of conformational changes accompanying desensitization in an ionotropic glutamate receptor. Cell.

[9] Messmore JM, Holmgren SK, Grilley JE, Raines RT (2000). Sulfur shuffle: Modulating enzymatic activity by thiol–disulfide interchange. Bioconjugate Chem.

[10] Park C, Raines RT (2001). Adjacent cysteine residues as a redox switch. Protein Eng.

[11] Grassetti DR, Murray JF (1967). Determination of sulfhydryl groups with 2,2′- or 4,4′-dithiopyridine. Arch. Biochem. Biophys.

[12] Riddles PW, Blakeley RL, Zerner B (1979). Ellman's reagent: 5,5′-Dithiobis(2-nitrobenzoic acid)—a reexamination. Anal. Biochem.

[13] Zalipsky S (1995). Functionalized poly(ethylene glycol) for preparation of biologically relevant conjugates. Bioconjugate Chem.

