# Supplementary material for: 1,9-Bis(2-pyridyl)-1,2,8,9-tetrathia-5-oxanonane
Source: Molbank. Author manuscript; Available in PMC 2010 May 12. (PMC2868194; doi:10.3390/M642)

Stabilization of the Collagen Triple Helix by *O*-Methylation of Hydroxyproline Residues

Frank W. Kotch, Ilia A. Guzei, and Ronald T. Raines*

Departments of Chemistry and Biochemistry, University of Wisconsin, Madison, Wisconsin 53706

| **Page** | **Contents** |
| --- | --- |
| S1 | Table of Contents |
| S2–S7 | Experimental Procedures |
| S8 | References |
| S9 | Table S-1: Crystal Data for Ac-Mop-OMe |
| S10 | Table S-2: Atomic Coordinates for Ac-Mop-OMe |
| S11–S12 | Table S-3: Bond Lengths and Angles for Ac-Mop-OMe |
| S13 | Table S-4: Displacement Parameters for Ac-Mop-OMe |
| S14 | Table S-5: Hydrogen Parameters for Ac-Mop-OMe |
| S15 | Table S-6: Torsion Angles for Ac-Mop-OMe |
| S16 | ^1^H NMR Spectrum of Boc-Mop-OH |
| S17 | ^13^C NMR Spectrum of Boc-Mop-OH |
| S18 | ^1^H NMR Spectrum of Boc-Mop-Gly-OBn |
| S19 | ^13^C NMR Spectrum of Boc-Mop-Gly-OBn |
| S20 | ^1^H NMR Spectrum of Fmoc-Pro-Mop-Gly-OBn |
| S21 | ^13^C NMR Spectrum of Fmoc-Pro-Mop-Gly-OBn |
| S22 | ^1^H NMR Spectrum of Fmoc-Pro-Mop-Gly-OH |
| S23 | ^13^C NMR Spectrum of Fmoc-Pro-Mop-Gly-OH |
| S24 | ^1^H NMR Spectrum of [^13^C]Ac-Mop-OMe |
| S25 | ^13^C NMR Spectrum of [^13^C]Ac-Mop-OMe |

**General Experimental.** Chemicals were from Aldrich or Acros (reagent grade or better), and were used without further purification. Amino acids were from Novabiochem, with the exception of Boc-Hyp-OH, which was from Chem-Impex International (Wood Dale, IL). (ProHypGly)_10_·10H_2_O was from Peptides International (Louisville, KY). Anhydrous DMF and CH_2_Cl_2_ were obtained from a CYCLE-TAINER® solvent delivery system from J. T. Baker (Phillipsburg, NJ). All other solvents were from Fisher Scientific (Pittsburgh, PA). Flash chromatography was performed with columns of silica gel 60, 230–400 mesh (Silicycle, Québec City, QC, Canada). Semi-preparative HPLC was performed with a Zorbax C8 reversed-phase column and analytical HPLC was performed with an Agilent C8 reversed-phase column. Linear gradients of solvent A (H_2_O with 0.1% v/v TFA) and solvent B (CH_3_CN with 0.1% v/v TFA) were used for HPLC analysis and purification.

The removal of solvents and other volatile materials “under reduced pressure” refers to the use of a rotary evaporator at water-aspirator pressure (<20 torr) and a water bath of <40 °C. Residual solvent was removed from samples at high vacuum (<0.1 torr). The term “high vacuum” refers to vacuum achieved by a mechanical belt-drive oil pump.

NMR spectra were recorded on either a Bruker DMX-400 Avance spectrometer or a Bruker DMX-500 Avance spectrometer at the National Magnetic Resonance Facility at Madison (NMRFAM). Some compounds with a carbamate protecting group exist as mixtures of rotomers that do not interconvert on the NMR time scale at ambient temperatures and therefore exhibit two sets of NMR signals (as indicated).

Mass spectrometry was performed with either a Micromass LCT (electrospray ionization, ESI) in the Mass Spectrometry Facility in the University of Wisconsin Department of Chemistry or an Applied Biosystems Voyager DE-Pro (matrix-assisted laser desorption/ionization, MALDI) mass spectrometer in the University of Wisconsin Biophysics Instrumentation Facility.

Fmoc-Pro-Mop-Gly-OH was synthesized in 6 steps (overall yield: 30%) by the route shown in Scheme S-1.

**Scheme S-1**


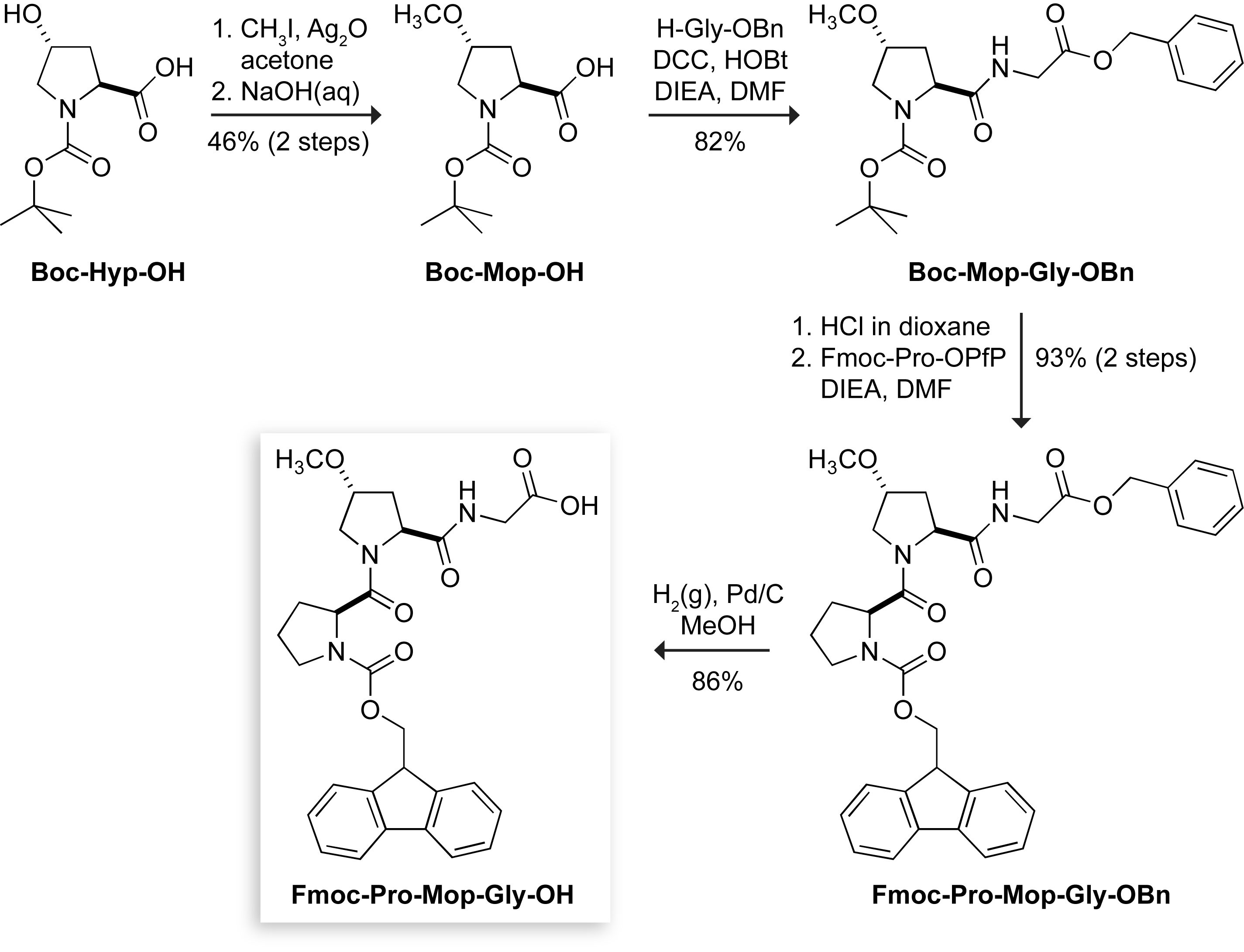


***N*-*tert*-Butoxycarbonyl-(2*S*,4*R*)-4-methoxyproline (Boc-Mop-OH).** This preparation is similar to that reported by Krapcho et al. for Cbz-Mop-OH.^S1^ To a solution of *N*-*tert*-Butoxycarbonyl-(2*S*,4*R*)-4-hydroxyproline (Boc-Hyp-OH, 2.00 g, 8.65 mmol) and CH_3_I (1.90 mL, 30.3 mmol) in acetone (50 mL) was added Ag_2_O (6.50 g, 28.1 mmol). The vessel was purged with Ar(g) and sealed with a septum, and the resulting suspension was stirred at room temperature for 24 h. The mixture was filtered and evaporated to a residue under reduced pressure. The residue was dried under high vacuum for 1 h, and then resubmitted to the reaction conditions a second time. After filtering and evaporating, the residue was dissolved in H_2_O (60 mL), a solution of NaOH (0.36 g, 9.08 mmol) in 1 mL H_2_O was added, and the solution was stirred at room temperature for 24 h (to hydrolyze any methyl ester that might have formed during alkylation). The aqueous mixture was acidified to pH 1 by the addition of 3 N HCl(aq) and extracted with CH_2_Cl_2_ (3 × 60 mL). The combined organic layers were dried over MgSO_4_(s) and evaporated under reduced pressure, and the product was then isolated by flash chromatography (100:0–95:5 CH_2_Cl_2_/MeOH) affording Boc-Mop-OH (0.97 g, 46%) as a tinted residue. ^1^H NMR (500 MHz, CDCl_3_, ~3:2 ratio of 2 rotamers): *δ* 4.44 (t, *J* = 7.5 Hz, 0.6 H), 4.34 (t, *J* = 7.9 Hz, 0.4 H), 4.01–3.95 (m, 1 H), 3.70–3.65 (m, 0.4 H), 3.60–3.52 (m, 1 H), 3.49 (dd, *J* = 11.7, 4.8 Hz, 0.6 H), 3.33 (overlapping s, 3 H), 2.46–2.36 (m, 1 H), 2.32–2.24 (m, 0.6 H), 2.17–2.07 (m, 0.4 H), 1.49 (s, 5.4 H), 1.43 (s, 3.6 H); ^13^C NMR (125 MHz, CDCl_3_, ~3:2 ratio of 2 rotamers): *δ* 178.9, 176.0, 156.1, 154.0, 81.7, 80.9, 78.4, 78.2, 57.9 (2 signals), 56.9 (2 signals), 51.8, 51.0, 36.4, 34.3, 28.5, 28.4; HRMS (ESI): *m*/*z* calcd for C_11_H_19_NO_5_Na ([M+Na]^+^) 268.1161, found 268.1151.

***N*-*tert*-Butoxycarbonyl-(2*S*,4*R*)-4-methoxyprolylglycine Benzyl Ester (Boc-Mop-Gly-OBn).** A solution of Boc-Mop-OH (0.42 g, 1.71 mmol), H-Gly-OBn·TsOH (0.64 g, 1.88 mmol), DCC (0.35 g, 1.71 mmol), HOBt·H_2_O (0.26 g, 1.71 mmol) and DIEA (0.89 mL, 5.13 mmol) in DMF (30 mL) was stirred at room temperature under Ar(g) for 20 h. The mixture was filtered to remove insoluble DCU and concentrated by rotary evaporation under high vacuum. The product was isolated by flash chromatography (two columns were needed; the first was run in 8:2 EtOAc/hexanes and the second in 7:3 EtOAc/hexanes) affording Boc-Mop-Gly-OBn (0.55 g, 82%) as a clear oil. ^1^H NMR (500 MHz, CDCl_3_, ~3:2 ratio of 2 rotamers): *δ* 7.41–7.32 (m, 5 H), 6.53 (app bs, 0.4 H), 5.22–5.15 (m, 2 H), 4.44 (dd, *J* = 7.9, 5.6 Hz, 0.6 H); 4.31 (t, *J* = 7.8 Hz, 0.4 H), 4.16–3.97 (m, 2.6 H), 3.93 (app bs, 0.4 H), 3.81–3.72 (m, 0.6 H), 3.53–3.43 (m, 1.4 H), 3.32 and 3.31 (overlapping s, 3 H), 2.49–2.42 (m, 0.6 H), 2.41–2.33 (m, 0.4 H), 2.16–2.05 (m, 1 H), 1.89 (bs, 0.6 H), 1.47 (s, 5 H), 1.42 (s, 4 H); ^13^C NMR (125 MHz, CDCl_3_, ~3:2 ratio of 2 rotamers): *δ* 173.0, 172.2, 169.8, 169.6, 156.0, 154.8, 135.3, 135.1, 128.8 (2 signals), 128.7, 128.6, 128.5, 81.1, 81.0, 78.7, 78.2, 67.5, 67.3, 60.0, 58.7, 57.0, 56.9, 51.7, 51.5, 41.6, 41.2, 36.8, 33.7, 28.5, 28.4; HRMS (ESI): *m*/*z* calcd for C_20_H_28_N_2_O_6_Na ([M+Na]^+^) 415.1845, found 415.1852.

***N*-9-Fluorenylmethoxycarbonyl-(2*S*)-prolyl-(2*S*,4*R*)-4-methoxyprolylglycine Benzyl Ester (Fmoc-Pro-Mop-Gly-OBn).** Boc-Mop-Gly-OBn (0.54 g, 1.38 mmol) was dissolved in 14 mL of 4N HCl in dioxane (56 mmol) and the solution was stirred at room temperature under Ar(g) for 2.5 h. The mixture was evaporated under reduced pressure, the remaining white solid (H-Mop-Gly-OBn·HCl) was azeotroped twice with toluene and dried under high vacuum for 2 h. The solid was dissolved in DMF (20 mL), FmocPro pentafluorophenyl ester (0.69 g, 1.38 mmol) and DIEA (0.48 mL, 2.75 mmol) were added, and the mixture was stirred at room temperature under Ar(g) for 20 h. The mixture was concentrated by rotary evaporation under high vacuum, and the product was isolated by flash chromatography (9:1 EtOAc/hexanes) affording Fmoc-Pro-Mop-Gly-OBn (0.78 g, 93% over 2 steps) as a clear residue. ^1^H NMR (400 MHz, CDCl_3_, mixture of 3 or more rotomers, integrations are approximate): *δ* 8.28 (t, *J* = 6.1 Hz, 0.3 H), 7.78–7.72 (m, 2 H), 7.65–7.51 (m, 2 H), 7.45–7.28 (m, 8 H), 7.27–7.23 (m, 1 H + CHCl_3_), 7.21–7.18 (m, 1 H), 5.13 (app dd, *J* = 22.4, 12.3 Hz, 1.3 H), 5.00 (app dd, *J* = 25.5, 12.3 Hz, 0.7 H), 4.76 (dd, *J* = 4.4, 4.0 Hz, 0.4 H), ), 4.63 (dd, *J* = 4.4, 4.0 Hz, 0.2 H), 4.55–4.30 (m, 3.2 H), 4.28–4.07 (m, 2.6 H), 4.07–3.86 (m, 2.2 H), 3.76–3.46 (m, 4 H), 3.39 (dd, *J* = 5.3, 5.0 Hz, 0.2 H), 3.34 (s, 1.3 H), 3.29 (s, 1 H), 3.21 (s, 0.6 H), 2.55–2.45 (m, 1 H), 2.44–2.36 (m, 0.4 H), 2.25–1.80 (m, 6 H); ^13^C NMR (100 MHz, CDCl_3_, mixture of 3 or more rotomers): *δ* 172.5 (2 signals), 172.1, 171.9, 171.5, 171.3, 169.6, 169.5, 169.3, 162.7, 155.5, 155.1, 154.6, 144.6, 144.3, 144.2, 144.0, 143.8, 141.5 (multiple signals), 135.4, 128.8, 128.7, 127.8 (multiple signals), 127.2, 125.4, 125.2, 125.1, 125.0, 120.1 (2 signals), 79.1, 79.0, 67.9, 67.6, 67.3, 67.2, 67.1, 59.6, 58.8, 58.6, 58.1, 58.0, 57.2, 57.1, 51.5, 51.0, 47.6, 47.4, 47.2 (2 signals), 46.9, 41.6, 41.5, 37.3, 32.5, 32.2, 30.5, 29.4 (2 signals), 24.8, 24.5, 23.3; HRMS (ESI): *m*/*z* calcd for C_35_H_37_N_3_O_7_Na ([M+Na]^+^) 634.2529, found 634.2517.

***N*-9-Fluorenylmethoxycarbonyl-(2*S*)-prolyl-(2*S*,4*R*)-4-methoxyprolylglycine (Fmoc-Pro-Mop-Gly-OH).** A suspension of Fmoc-Pro-Mop-Gly-OBn (0.74 g, 1.21 mmol) and Pd/C (0.07 g) in MeOH (25 mL) was stirred under an atmosphere of H_2_(g) for 2 h. The mixture was filtered through a pad of Celite® and evaporated leaving a white solid. The product was isolated by flash chromatography (9:1 CH_2_Cl_2_/MeOH–EtOH) affording Fmoc-Pro-Mop-Gly-OH (0.54 g, 86%) as a white solid. ^1^H NMR (500 MHz, DMSO-*d*_6_, ~1:1 ratio of 2 rotamers): *δ* 7.91–7.82 (m, 2 H), 7.65 (t, *J* = 7.4 Hz, 1 H), 7.58 (d, *J* = 7.4 Hz, 0.5 H), 7.55 (d, *J* = 7.5 Hz, 0.5 H), 7.44–7.28 (m, 5 H), 4.60 (dd, *J* = 8.6, 3.1 Hz, 0.5 H), 4.52 (dd, *J* = 8.6, 2.7 Hz, 0.5 H), 4.45–4.40 (m, 0.5 H), 4.34 (app t, *J* = 7.4 Hz, 0.5 H), 4.27–4.10 (m, 3 H), 4.04–3.97 (m, 1 H), 3.76–3.70 (m, 0.5 H), 3.67–3.57 (m, 1.5 H), 3.42–3.29 (signals under H_2_O peak), 3.22 (s, 1.5 H), 3.11 (s, 1.5 H), 2.32–2.29 (m, 0.5 H), 2.28–2.18 (m, 0.5 H), 2.17–2.06 (m, 1.5 H), 2.02–1.73 (m, 4.5 H); ^13^C NMR (125 MHz, DMSO-*d*_6_, ~1:1 ratio of 2 rotamers): *δ* 171.7, 171.0, 170.3, 170.2, 153.8, 143.9 (3 signals), 143.8, 140.7, 140.6, 127.7 (2 signals), 127.2, 127.1, 125.3, 125.1, 120.1, 120.0 (2 signals), 78.6, 78.4, 66.8, 66.5, 58.3 (2 signals), 57.9, 57.6, 56.1, 56.0, 55.9, 51.3, 51.2, 47.0, 46.7, 46.6, 46.3, 42.0, 34.3, 29.6, 28.6, 23.7, 22.6; HRMS (ESI): *m*/*z* calcd for C_28_H_31_N_3_O_7_Na ([M+Na]^+^) 544.2060, found 544.2039.

***N*-(2-^13^CH_3_-Acetyl)-(2*S*,4*R*)-4-methoxyproline methyl ester (2-^13^CH_3_-Ac-Mop-OMe).** Following the method of Nudelman et al.,^S2^ a solution of Boc-Mop-OH (100 mg, 0.41 mmol) in anhydrous MeOH (12.5 mL) was cooled to 0 °C. Acetyl chloride (12.5 mL) was added dropwise over 5 min., the mixture was allowed to warm to room temperature and stirred under Ar(g) for 7 h. The solvent was evaporated under reduced pressure, and the resulting residue (H-Mop-OMe·HCl) was dried under high vacuum for 1 h. The residue was dissolved in CH_2_Cl_2_ (20 mL), *N,N*-Dimethylaminopyridine (500 mg, 3.84 mmol) was added, followed by dropwise addition of H_3_^13^CC(O)Cl (273 mL, 3.84 mmol) and the mixture was stirred at room temperature under Ar(g) for 24 h. MeOH (5 mL) was added to quench the reaction. The mixture was concentrated under reduced pressure, the remaining residue was dissolved in CH_2_Cl_2_ (40 mL) and washed with 10% w/v aqueous citric acid. The aqueous layer was back-extracted with CH_2_Cl_2_ (3x 20 mL), and the combined organic layers were dried over MgSO_4_(s) and evaporated under reduced pressure. The product was isolated by flash chromatography (1:1 EtOAc/hexanes to elute byproducts followed by 94:6 EtOAc/MeOH) affording 2-^13^CH_3_-Ac-Mop-OMe (71 mg, 86%) as a white solid. ^1^H NMR (500 MHz, CDCl_3_, ~4:1 ratio of *trans*:*cis* amide bond rotomers): *δ* 4.50 (app t, *J* = 7.8 Hz, 1 H), 4.11–4.07 (m, 0.8 H), 4.02–3.98 (m, 0.2 H), 3.96–3.91 (m, 0.2 H), 3.82–3.78 (m, 0.8 H), 3.79 (s, 0.6 H), 3.75 (s, 2.4 H), 3.58–3.51 (m, 1 H), 3.35 (s, 2.4 H), 3.32 (s, 0.6 H), 2.52–2.45 (m, 0.2 H), 2.40–2.32 (m, 0.8 H), 2.27–2.19 (m, 0.2 H), 2.11–2.03 (m, 0.8 H), 2.10 (d, *J*_C–H_ = 128 Hz, 2.4 H), 1.97 (d, *J*_C–H_ = 128 Hz, 0.6 H); ^13^C NMR (125 MHz, CDCl_3_, ~4:1 ratio of *trans*:*cis* amide bond rotomers): *δ* 173.0, 172.8, 170.2 (d, *J* = 51 Hz), 169.6 (d, *J* = 52 Hz), 79.0, 77.4, 58.8, 57.4, 56.9, 52.9, 52.5, 50.8, 37.3, 34.5, 22.5 and 21.9 (labeled ^13^C); HRMS (ESI): *m*/*z* calcd for C_8_^13^CH_15_NO_4_Na ([M+Na]^+^) 225.0932, found 225.0925.

**Attachment of Fmoc-Pro-Mop-Gly-OH onto 2-chlorotrityl chloride resin.** Under Ar(g), 69 mg (0.110 mmol) of 2-chlorotrityl chloride resin (loading 1.6 mmol/g) was swollen in dry CH_2_Cl_2_ (0.4 mL). A solution of Fmoc-Pro-Mop-Gly-OH (40 mg, 0.077 mmol) in dry CH_2_Cl_2_ (1.0 mL) was added followed by DIEA (58 μL, 0.330 mmol), and the resin suspension was agitated gently for 2.5 h. Anhydrous CH_3_OH (0.2 mL) was added to the mixture and the suspension was agitated for an additional 15 min (to cap any remaining active sites on the resin). The resin-bound peptide was isolated by gravity filtration, washed with dry CH_2_Cl_2_ (20 x 1 mL), and dried over KOH under high vacuum for 18 h. Loading was measured by UV spectroscopy using the reported protocol^S3^ to be 0.57 mmol/g.

**Peptide Synthesis.** (Pro-Mop-Gly)_10_ was synthesized with free N- and C-termini by segment condensation of Fmoc-Pro-Mop-Gly-OH on solid phase using an Applied Biosystems Synergy 432A Peptide Synthesizer at the University of Wisconsin–Madison Biotechnology Center. The first trimer was loaded onto the resin as described above. Fmoc-deprotection was achieved by treatment with 20% v/v piperidine in DMF. The Fmoc-tripeptides (3 equiv) were converted to active esters by treatment with HBTU, DIEA, and HOBt. Couplings were allowed to proceed for 45–60 min at room temperature. The peptide was cleaved from the resin in 38:1:1 TFA/H_2_O/triisopropylsilane (1 mL), precipitated from *tert*-butylmethylether at 0 °C, and isolated by centrifugation. Semi-preparative HPLC was used to purify (Pro-Mop-Gly)_10_ (gradient: 10% B to 30% B over 60 min). The peptide was >90% pure by analytical HPLC and characterized by MALDI–TOF mass spectrometry: (*m*/*z*) [M + H]^+^ calcd for (Pro-Mop-Gly)_10_ 2830.4, found 2831.3.

**Circular Dichroism (CD) Spectroscopy.** CD spectra were recorded with an Aviv 202SF circular dichroism spectrometer. Spectra were recorded on peptide solutions (100 μM in 50 mM HOAc (pH 2.9)) that had been incubated at ≤4 °C for ≥48 h in 1-nm increments with a 3-s averaging time, 1‑nm bandpass, and 0.1-cm pathlength. Samples for thermal stability experiments were generated by incubating peptide solutions (200 µM) at 4 °C for ≥48 h. The solutions were then heated from 4 °C to 97 °C (for samples in 50 mM HOAc (aq) (pH 2.9)) or to 106 °C (for samples in 2:1 ethylene glycol/50 mM HOAc(aq)) at 3-°C increments with a 5-min equilibration at each step. The ellipticity at 225 nm was monitored with a 5-s averaging time, 1‑nm bandpass, and 0.1-cm pathlength. Values of *T*_m_ were determined by fitting the data to a two-state model.^S4^

**Differential Scanning Calorimetry (DSC).** DSC measurements were conducted on a VP‑DSC instrument (MicroCal, LLC, Northampton, MA). For each peptide, an instrument baseline was established by filling both the sample and the reference cell with degassed 50 mM HOAc(aq) (pH 2.9) and scanning from 5–98 °C at 15 °C/h until at least 3 consecutive overlaying scans were observed. The last of these scans was used as the baseline for each subsequent peptide scan.

Peptide solutions (~0.5 mg/mL in 50 mM HOAc(aq)), incubated at 4 °C for ≥48 h, were degassed and loaded into the sample cell (without removing the reference solution) during the cool down from the final baseline scan (at ~15 °C). Samples were scanned from 5–98 °C at 15 °C/h; the first scan of each sample was used in the analysis. Subsequent scans of the same sample showed a decreased melting enthalpy indicative of incomplete recovery of triple helix (60–80%); because complete helical folding of these peptides requires a few hours at low temperature, and the sample cells were cooled at ~15 °C/min, incomplete folding is expected during the rapid cool down cycle. Nevertheless, a second run with a fresh sample (incubated at 4 °C for ≥48 h) gave a trace that overlaid with the initial scan for both peptides.

After DSC measurements, peptide concentrations were determined by quantitative amino acid analysis (Scientific Research Consortium, Inc., St. Paul, MN). Peptide concentrations of 231 μM for (ProHypGly)_10_ and 129 μM for (ProMopGly)_10_ were calculated from the average of Gly and Pro content.

Data processing was done using the MicroCal software in the Origin 7 program (OriginLab Corp., Northampton, MA). For each sample, the appropriate reference scan was subtracted from the sample scan and the data were normalized to the monomer concentrations (determined above). A progress baseline was then subtracted from the data, giving the traces shown in Figure 1C of the manuscript.

Values of Δ*H* (per mole of monomer) were obtained by direct integration of the DSC exotherms. Using Δ*H* and the exotherm maxima as the *T*_m_ (64.3 °C for (ProHypGly)_10_ and 73.8 °C for (ProMopGly)_10_), *T*Δ*S* (at *T*_m_) was calculated with the equation: *T*_m_ = Δ*H*/(Δ*S* + *R*·ln(0.75*c*^2^)), where *c* is the concentration of monomeric peptide.^S5^ Values of Δ*G* were then calculated with the equation: Δ*G* = Δ*H* – *T*Δ*S*. Table 1 in the manuscript lists only data for (ProMopGly)_10_, comparing these data to those reported by Kobayashi and coworkers^S6^ for (ProHypGly)_10_ and (ProFlpGly)_10_. The data of Kobayashi and coworkers were normalized to *T*° = 71.9 °C (which is the *T*° for (ProProGly)_10_, *i*.*e*. where Δ*G* = 0). Because our thermodynamic data for (ProMopGly)_10_ were determined at nearly the same temperature (73.8 °C), comparisons of the three peptides are meaningful.

The values for (ProHypGly)_10_, however, were determined at 64.3 °C, and such comparisons become unsound with larger differences in temperature. For (ProHypGly)_10_, the thermodynamic parameters at 64.3 °C were Δ*H* = –29.5 kcal/mol, *T*Δ*S* = –27.3 kcal/mol, and Δ*G* = –2.2 kcal/mol. Note that Δ*G* agrees with that reported by Kobayashi and coworkers (–2.0 kcal/mol).

**Measurement of the Amide Bond *trans*/*cis* Equilibrium Constant (*K*_t/c_) in [^13^CH_3_]Ac-Mop-OMe.** [^13^CH_3_]Ac-Mop-OMe (10 mg) was dissolved in 94:6 D_2_O/CH_3_OD (850 μL). The ^13^C NMR spectrum was recorded using an inverse gated decoupling pulse program with a relaxation delay of 60 s and a pulse width of 10 μs. A total of 32 transients were collected. The spectral baseline was corrected and peaks corresponding to the labeled carbon were integrated with the software package NUTS.^S7^ Values of *K*_t/c_ were determined by the relative areas of the *trans* and *cis* peaks for the labeled carbon.

**Crystallization of Ac-Mop-OMe.** [^13^CH_3_]Ac-Mop-OMe (10 mg) was dissolved in 1:1 CH_2_Cl_2_/hexanes (1 mL) and the solution was allowed to stand at room temperature in a loosely-capped vial. Slow evaporation afforded crystals suitable for X-ray analysis after ~72 h (all solvent had evaporated).

**Crystallographic Data Collection.** A colorless crystal with approximate dimensions 0.49 × 0.41 × 0.23 mm^3^ was selected under oil under ambient conditions and attached to the tip of a nylon loop. The crystal was mounted in a stream of cold N_2_(g) at 100(2) K and centered in the X-ray beam by using a video camera.

The crystal evaluation and data collection were performed on a Bruker CCD-1000 diffractometer with Mo K_α_ (*λ* = 0.71073 Å) radiation and the diffractometer-to-crystal distance of 4.9 cm.

The initial cell constants were obtained from three series of scans at different starting angles. Each series consisted of 20 frames collected at intervals of 0.3° in a 6° range about *ω* with the exposure time of 10 s per frame. A total of 69 reflections were obtained. The reflections were indexed successfully by an automated indexing routine built in the SMART program. The final cell constants were calculated from a set of 4805 strong reflections from the actual data collection.

Data were collected by using the full-sphere data collection routine. The reciprocal space was surveyed to the extent of a full sphere to a resolution of 0.80 Å. A total of 6155 data were harvested by collecting three sets of frames with 0.3° scans in *ω* and *φ* with an exposure time 10 s per frame. These highly redundant datasets were corrected for Lorentz and polarization effects. The absorption correction was based on fitting a function to the empirical transmission surface as sampled by multiple equivalent measurements.^S8^

**Crystallographic Structure Solution and Refinement.** The systematic absences in the diffraction data were consistent for the space groups *P*2_1_ and *P*2_1_/*m*. The *E*-statistics strongly suggested the non-centrosymmetric space group *P*2_1_ that yielded chemically reasonable and computationally stable results of refinement.^S8^

A successful solution by the direct methods provided most non-hydrogen atoms from the *E*‑map. The remaining non-hydrogen atoms were located in an alternating series of least-squares cycles and difference Fourier maps. All non-hydrogen atoms were refined with anisotropic displacement coefficients. All hydrogen atoms were found in the difference map and refined independently. The absolute configuration was assigned from the known synthetic procedure.

The final least-squares refinement of 187 parameters against 2089 data resulted in residuals *R* (based on *F*^2^ for *I* ≥ 2*σ*) and *wR* (based on *F*^2^ for all data) of 0.0273 and 0.0706, respectively. The final difference Fourier map was featureless.

The molecular diagram is drawn with 50% probability ellipsoids.

**References**

(S1) Krapcho, J. et al. *J. Med. Chem.* **1988**, *31*, 1148–1160.

(S2) Nudelman, A.; Bechor, Y.; Falb, E.; Fischer, B.; Wexler, B. A.; Nudelman, A. *Synth. Commun.* **1998**, *28*, 471–474.

(S3) Applied Biosystems *Determination of the Amino Acid Substitution Level via an Fmoc Assay*; Technical Note 123485 Rev 2; Documents on Demand–Applied Biosystems Web Page, http://docs.appliedbiosystems.com/search.taf (November 30, 2005).

(S4) Becktel, W. J.; Schellman, J. A. *Biopolymers* **1987**, *26*, 1859–1877.

(S5) Engel, J.; Bächinger, H. P. *Top. Curr. Chem.* **2005**, *247*, 7–33.

(S6) Nishi, Y.; Uchiyama, S.; Doi, M.; Nishiuchi, Y.; Nakazawa, T.; Ohkuba, T.; Kobayashi, Y. *Biochemistry* **2005**, *44*, 6034–6042.

(S7) NUTS–NMR Utility Transform Software, Acorn NMR, Inc., 7670 Las Positas Road, Livermore, CA 94551.

(S8) Bruker-AXS. (2000–2003) SADABS V.2.05, SAINT V.6.22, SHELXTL V.6.10 & SMART 5.622 Software Reference Manuals. Bruker-AXS, Madison, Wisconsin, USA.

***Table S-1*.** Crystal Data and Structure Refinement for Ac-Mop-OMe

__________________________________________________________________________

Identification code raines07

Empirical formula C_9_H_15_NO_4_

Formula weight 201.22

Temperature 100(2) K

Wavelength 0.71073 Å

Crystal system Monoclinic

Space group P2_1_

Unit cell dimensions *a* = 5.8595(7) Å *α* = 90°

*b* = 13.4363(16) Å *β* = 98.802(2)°

*c* = 6.5774(8) Å *γ* = 90°

Volume 511.74(11) Å^3^

*Z* 2

Density (calculated) 1.306 Mg/m^3^

Absorption coefficient 0.103 mm^–1^

*F*(000) 216

Crystal size 0.49 × 0.41 × 0.23 mm^3^

Theta range for data collection 3.03 to 26.41°

Index ranges –7 ≤ *h* ≤ 7, –16 ≤ *k* ≤ 16, –8 ≤ *l* ≤ 8

Reflections collected 6155

Independent reflections 2089 [*R*_int_ = 0.0187]

Completeness to theta = 26.41° 99.9%

Absorption correction Multi-scan with SADABS

Max. and min. transmission 0.9768 and 0.9515

Refinement method Full-matrix least-squares on *F*^2^

Data / restraints / parameters 2089 / 1 / 187

Goodness-of-fit on *F*^2^ 1.038

Final *R* indices [*I* > 2*σ*(*I*)] *R*_1_ = 0.0273, *wR*_2_ = 0.0700

*R* indices (all data) *R*_1_ = 0.0279, *wR*_2_ = 0.0706

Absolute structure parameter Assigned from synthesis

Largest diff. peak and hole 0.213 and –0.144 e.Å^–3^

_______________________________________________________________________

***Table S-2*.** Atomic Coordinates (×10^4^) and Equivalent Isotropic Displacement Parameters (Å^2^ × 10^3^) for Ac-Mop-OMe

________________________________________________________________________________

*x y* z *U*_eq_^a^

________________________________________________________________________________

O(1) 10655(2) 6883(1) 939(2) 23(1)

O(2) 8162(2) 5980(1) -3279(1) 25(1)

O(3) 11278(1) 4985(1) -2570(1) 18(1)

O(4) 6862(2) 3977(1) 3304(1) 23(1)

N(1) 7697(2) 5826(1) 968(2) 17(1)

C(1) 8741(2) 6708(1) 1419(2) 18(1)

C(2) 7454(2) 7452(1) 2526(2) 22(1)

C(3) 8812(2) 5058(1) -90(2) 16(1)

C(4) 9347(2) 5422(1) -2146(2) 17(1)

C(5) 11860(3) 5185(1) -4590(2) 24(1)

C(6) 7016(2) 4215(1) -386(2) 19(1)

C(7) 5668(2) 4353(1) 1412(2) 20(1)

C(8) 6832(3) 2915(1) 3420(3) 32(1)

C(9) 5545(2) 5481(1) 1635(2) 19(1)

________________________________________________________________________________

^a^*U*_eq_ is defined as 1/3 of the trace of the orthogonalized *U^ij^* tensor.

***Table S-3*.** Bond Lengths [Å] and Angles [°] for Ac-Mop-OMe

_________________________________________________

O(1)-C(1) 1.2333(15)

O(2)-C(4) 1.2002(16)

O(3)-C(4) 1.3415(15)

O(3)-C(5) 1.4465(15)

O(4)-C(7) 1.4237(16)

O(4)-C(8) 1.4283(17)

N(1)-C(1) 1.3460(16)

N(1)-C(3) 1.4540(15)

N(1)-C(9) 1.4721(15)

C(1)-C(2) 1.5054(17)

C(2)-H(2A) 0.961(19)

C(2)-H(2B) 0.968(18)

C(2)-H(2C) 0.94(2)

C(3)-C(4) 1.5154(16)

C(3)-C(6) 1.5388(17)

C(3)-H(3) 0.958(14)

C(5)-H(5A) 0.93(3)

C(5)-H(5B) 0.966(19)

C(5)-H(5C) 0.952(17)

C(6)-C(7) 1.5310(17)

C(6)-H(6A) 0.961(18)

C(6)-H(6B) 0.947(17)

C(7)-C(9) 1.5256(17)

C(7)-H(7) 0.968(15)

C(8)-H(8A) 0.98(2)

C(8)-H(8B) 0.94(2)

C(8)-H(8C) 0.97(2)

C(9)-H(9A) 1.007(17)

C(9)-H(9B) 0.935(17)

C(4)-O(3)-C(5) 115.76(10)

C(7)-O(4)-C(8) 113.15(11)

C(1)-N(1)-C(3) 120.53(9)

C(1)-N(1)-C(9) 126.33(10)

C(3)-N(1)-C(9) 112.81(10)

O(1)-C(1)-N(1) 120.75(11)

O(1)-C(1)-C(2) 122.68(12)

N(1)-C(1)-C(2) 116.57(10)

C(1)-C(2)-H(2A) 108.6(11)

C(1)-C(2)-H(2B) 110.2(11)

H(2A)-C(2)-H(2B) 107.8(15)

C(1)-C(2)-H(2C) 110.0(11)

H(2A)-C(2)-H(2C) 111.4(16)

H(2B)-C(2)-H(2C) 108.8(15)

N(1)-C(3)-C(4) 111.56(10)

N(1)-C(3)-C(6) 103.56(9)

C(4)-C(3)-C(6) 110.83(10)

N(1)-C(3)-H(3) 110.9(8)

C(4)-C(3)-H(3) 108.0(8)

C(6)-C(3)-H(3) 112.1(9)

O(2)-C(4)-O(3) 124.79(12)

O(2)-C(4)-C(3) 125.23(11)

O(3)-C(4)-C(3) 109.89(10)

O(3)-C(5)-H(5A) 110.8(14)

O(3)-C(5)-H(5B) 107.8(10)

H(5A)-C(5)-H(5B) 108.4(17)

O(3)-C(5)-H(5C) 108.6(10)

H(5A)-C(5)-H(5C) 110.9(18)

H(5B)-C(5)-H(5C) 110.3(15)

C(7)-C(6)-C(3) 103.67(10)

C(7)-C(6)-H(6A) 111.2(10)

C(3)-C(6)-H(6A) 111.5(10)

C(7)-C(6)-H(6B) 107.2(10)

C(3)-C(6)-H(6B) 109.7(10)

H(6A)-C(6)-H(6B) 113.0(13)

O(4)-C(7)-C(9) 107.05(11)

O(4)-C(7)-C(6) 112.93(10)

C(9)-C(7)-C(6) 103.45(10)

O(4)-C(7)-H(7) 106.7(9)

C(9)-C(7)-H(7) 113.7(9)

C(6)-C(7)-H(7) 113.1(9)

O(4)-C(8)-H(8A) 111.9(13)

O(4)-C(8)-H(8B) 112.7(13)

H(8A)-C(8)-H(8B) 104.6(17)

O(4)-C(8)-H(8C) 108.0(12)

H(8A)-C(8)-H(8C) 107.0(17)

H(8B)-C(8)-H(8C) 112.6(17)

N(1)-C(9)-C(7) 103.22(10)

N(1)-C(9)-H(9A) 111.7(10)

C(7)-C(9)-H(9A) 110.2(10)

N(1)-C(9)-H(9B) 111.5(10)

C(7)-C(9)-H(9B) 108.9(10)

H(9A)-C(9)-H(9B) 111.0(14)

_____________________________________________________________

***Table S-4*.** Anisotropic Displacement Parameters (Å^2^ × 10^3^) for Ac-Mop-OMe^a^

______________________________________________________________________________

*U*^11^ *U*^22^ *U*^33^ *U*^23^ *U*^13^ *U*^12^

______________________________________________________________________________

O(1) 22(1) 19(1) 30(1) 0(1) 9(1) -4(1)

O(2) 30(1) 26(1) 20(1) 4(1) 4(1) 9(1)

O(3) 19(1) 21(1) 16(1) 2(1) 6(1) 2(1)

O(4) 26(1) 19(1) 24(1) 3(1) 6(1) 1(1)

N(1) 15(1) 17(1) 18(1) -1(1) 5(1) 1(1)

C(1) 20(1) 16(1) 16(1) 3(1) 2(1) 1(1)

C(2) 28(1) 17(1) 23(1) 0(1) 7(1) 2(1)

C(3) 15(1) 16(1) 18(1) 0(1) 2(1) 1(1)

C(4) 18(1) 16(1) 18(1) -2(1) 2(1) -1(1)

C(5) 27(1) 29(1) 18(1) 3(1) 9(1) 2(1)

C(6) 18(1) 16(1) 21(1) -2(1) 2(1) -2(1)

C(7) 15(1) 19(1) 25(1) 1(1) 5(1) 0(1)

C(8) 43(1) 21(1) 35(1) 6(1) 12(1) 3(1)

C(9) 16(1) 19(1) 25(1) -1(1) 7(1) 0(1)

______________________________________________________________________________

^a^The anisotropic displacement factor exponent takes the form: –2*p^2^*[*h*^2^ a*^2^U^11^ + ... + 2 *h* *k* *a** *b** *U*^12^].

***Table S-5*.** Hydrogen Coordinates (×10^4^) and Isotropic Displacement Parameters (Å^2^ × 10^3^)

for Ac-Mop-OMe

________________________________________________________________________________

*x y z U*_eq_

________________________________________________________________________________

H(2A) 8130(30) 8097(14) 2420(30) 27(4)

H(2B) 7600(30) 7286(14) 3970(30) 30(4)

H(2C) 5880(40) 7451(14) 1960(30) 27(4)

H(3) 10230(20) 4851(11) 720(20) 9(3)

H(5A) 12190(40) 5858(19) -4730(30) 48(6)

H(5B) 13230(30) 4803(14) -4730(30) 27(4)

H(5C) 10600(30) 4983(15) -5600(30) 25(4)

H(6A) 7740(30) 3572(14) -340(20) 22(4)

H(6B) 5970(30) 4322(13) -1610(30) 18(4)

H(7) 4170(30) 4032(11) 1190(20) 13(3)

H(8A) 7810(40) 2608(17) 2510(30) 44(5)

H(8B) 5360(40) 2648(15) 2990(30) 36(5)

H(8C) 7470(30) 2724(15) 4820(30) 33(5)

H(9A) 4140(30) 5749(13) 730(30) 23(4)

H(9B) 5510(30) 5637(12) 3020(30) 19(4)

________________________________________________________________________________

***Table S-6*.** Torsion angles [°] for Ac-Mop-OMe

________________________________________________________________

C(3)-N(1)-C(1)-O(1) 0.20(17)

C(9)-N(1)-C(1)-O(1) 173.14(11)

C(3)-N(1)-C(1)-C(2) -179.69(10)

C(9)-N(1)-C(1)-C(2) -6.75(17)

C(1)-N(1)-C(3)-C(4) -58.09(14)

C(9)-N(1)-C(3)-C(4) 128.08(11)

C(1)-N(1)-C(3)-C(6) -177.34(10)

C(9)-N(1)-C(3)-C(6) 8.82(13)

C(5)-O(3)-C(4)-O(2) -2.82(19)

C(5)-O(3)-C(4)-C(3) 173.71(10)

N(1)-C(3)-C(4)-O(2) -35.67(16)

C(6)-C(3)-C(4)-O(2) 79.17(15)

N(1)-C(3)-C(4)-O(3) 147.82(10)

C(6)-C(3)-C(4)-O(3) -97.34(11)

N(1)-C(3)-C(6)-C(7) -27.98(12)

C(4)-C(3)-C(6)-C(7) -147.73(10)

C(8)-O(4)-C(7)-C(9) 171.94(11)

C(8)-O(4)-C(7)-C(6) -74.87(14)

C(3)-C(6)-C(7)-O(4) -78.66(12)

C(3)-C(6)-C(7)-C(9) 36.72(12)

C(1)-N(1)-C(9)-C(7) -159.42(12)

C(3)-N(1)-C(9)-C(7) 13.99(13)

O(4)-C(7)-C(9)-N(1) 88.55(11)

C(6)-C(7)-C(9)-N(1) -30.94(12)

________________________________________________________________


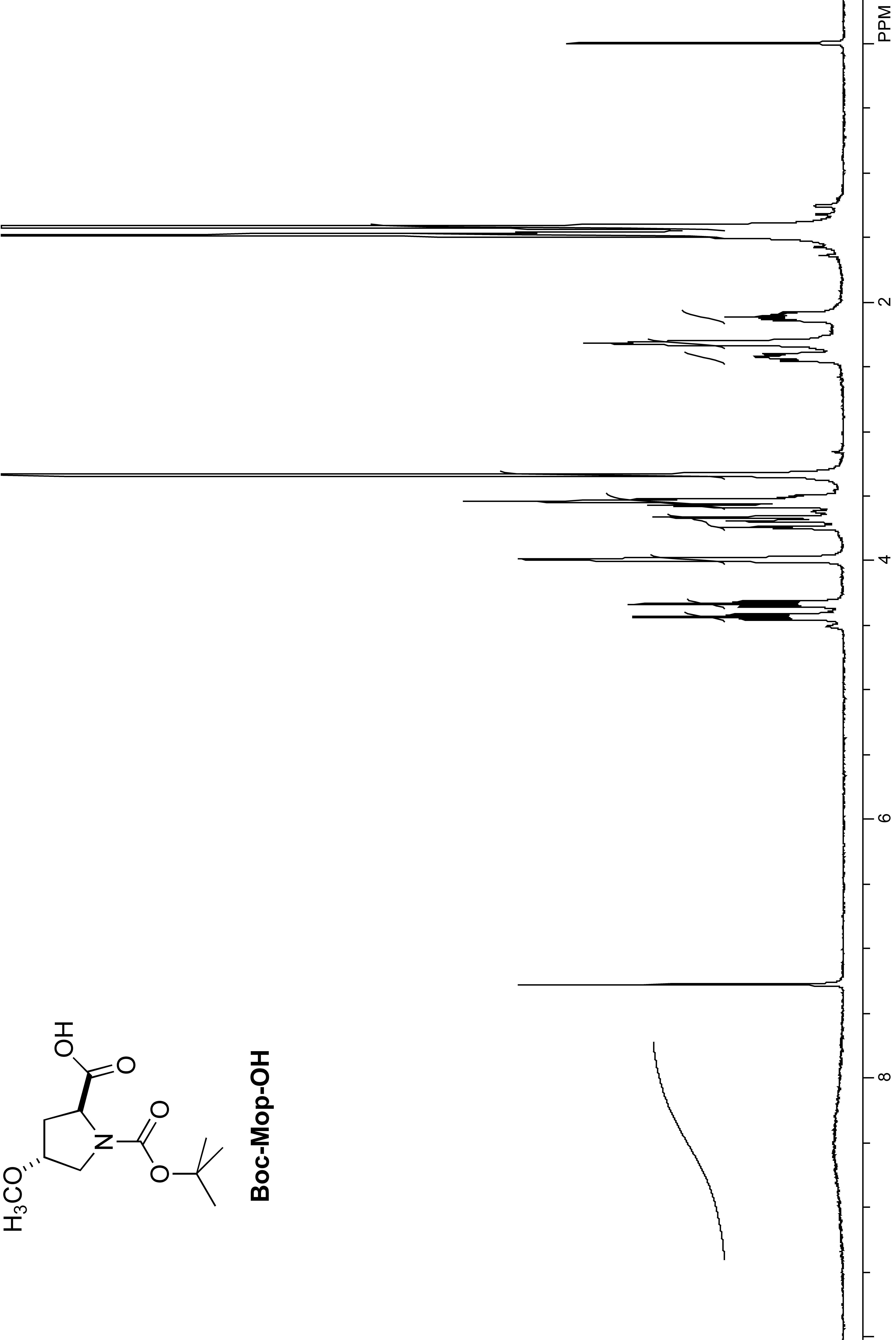


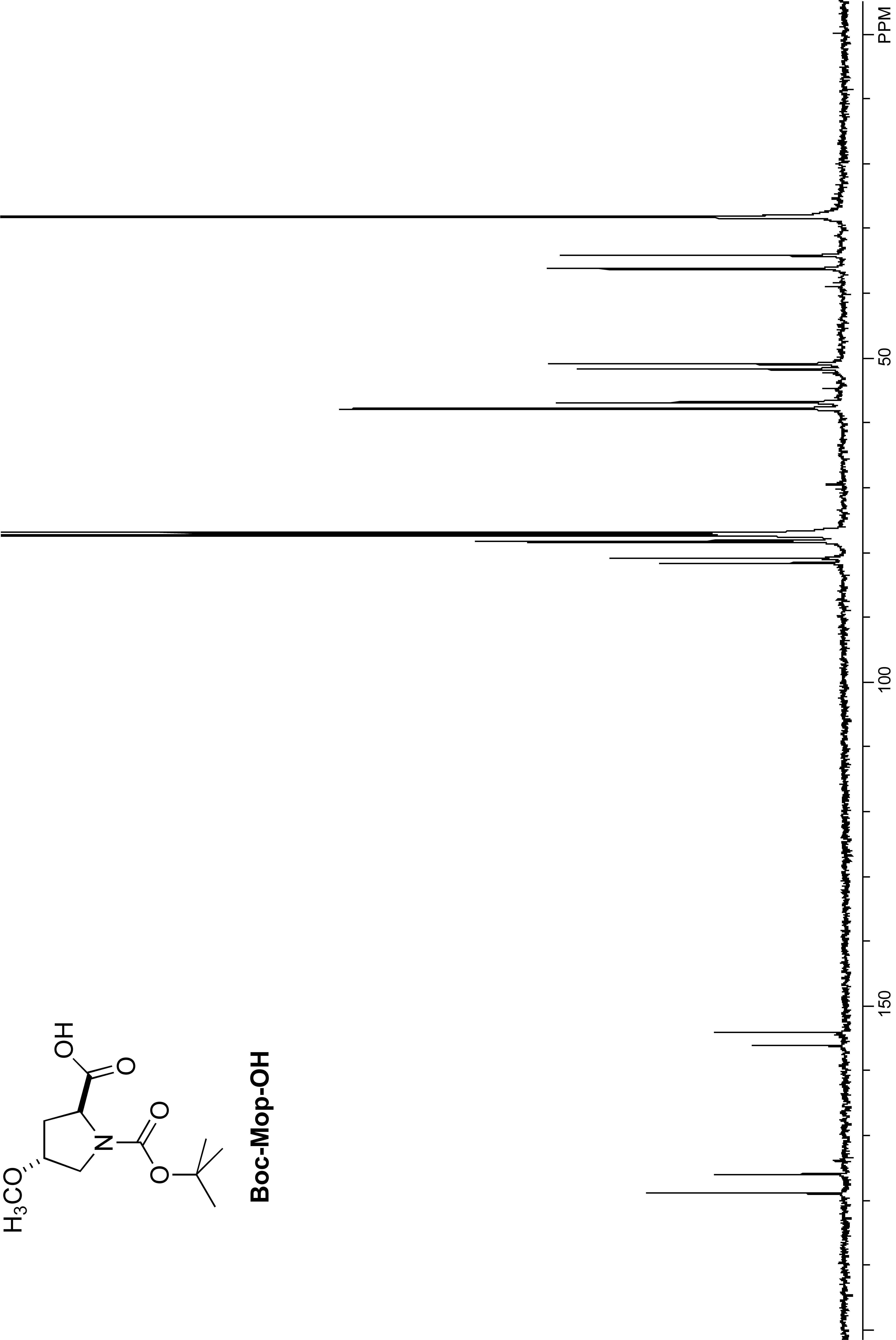


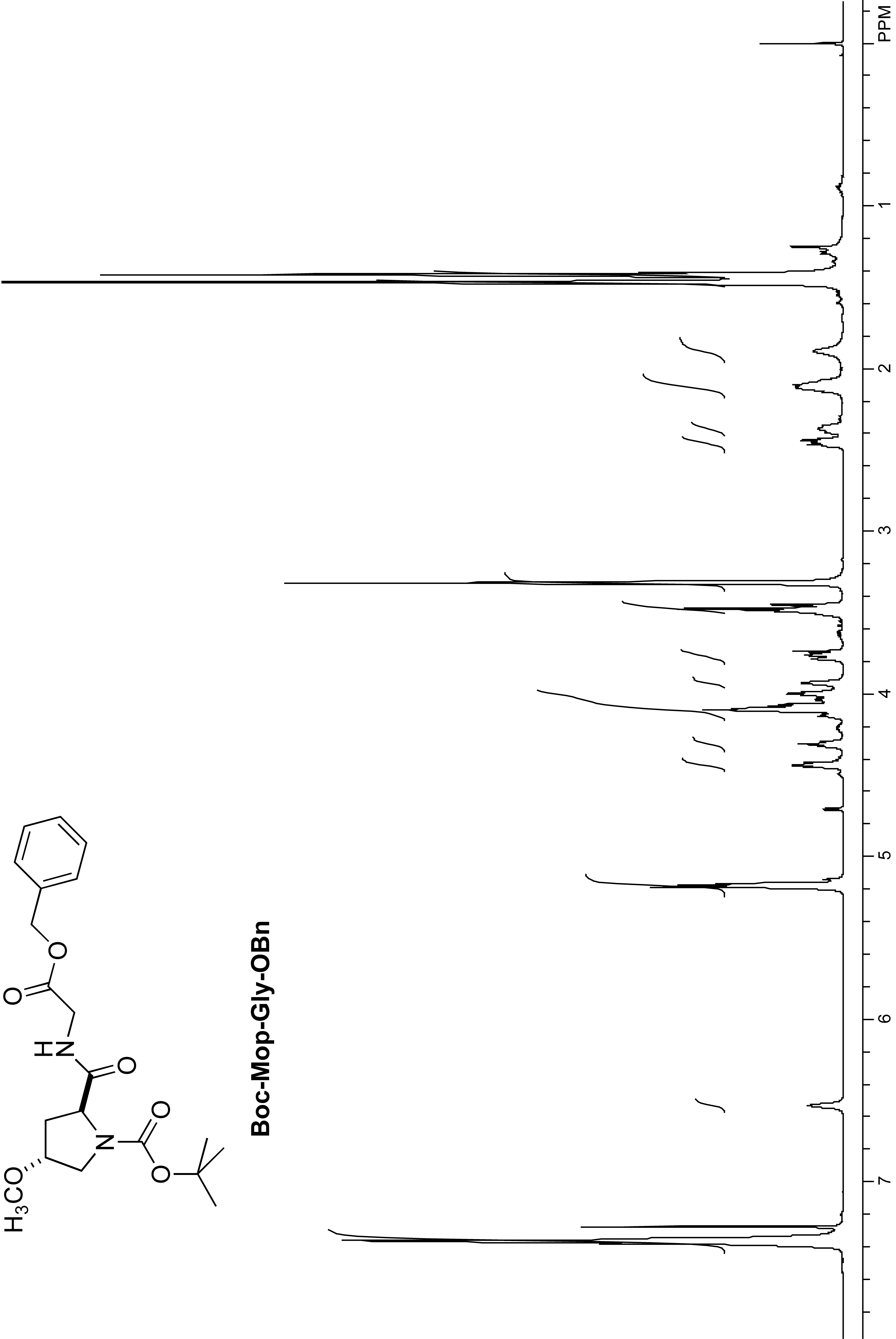

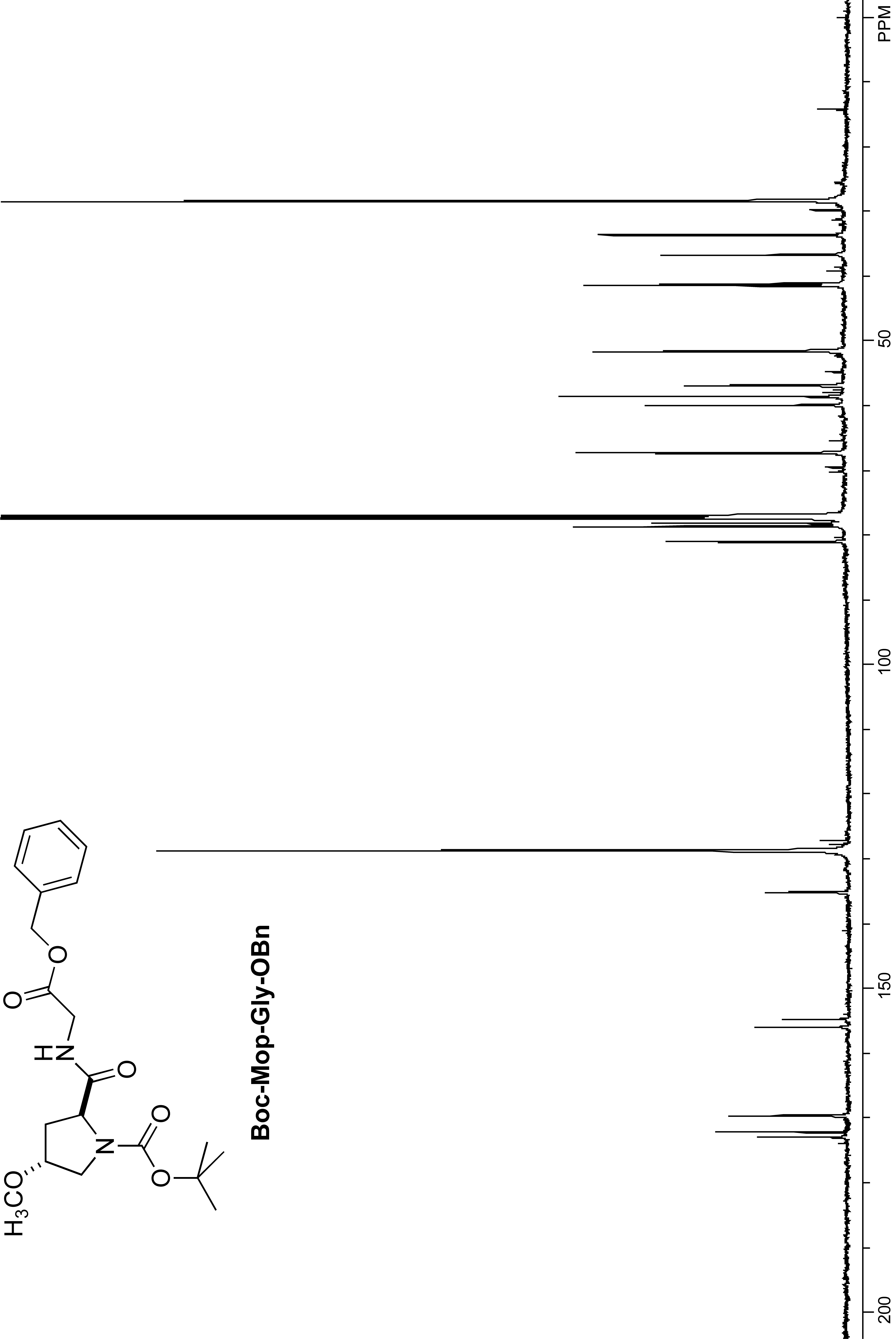


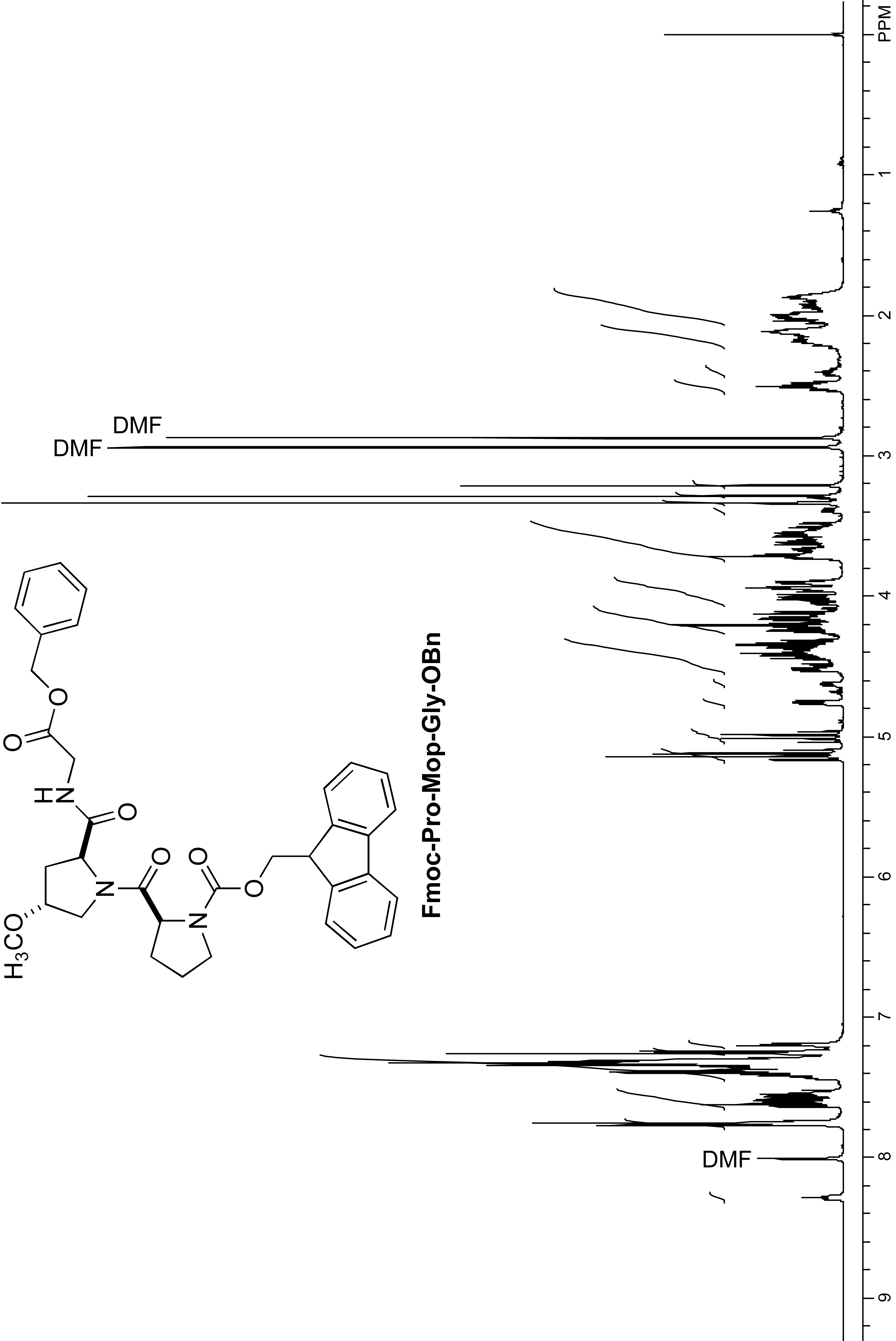


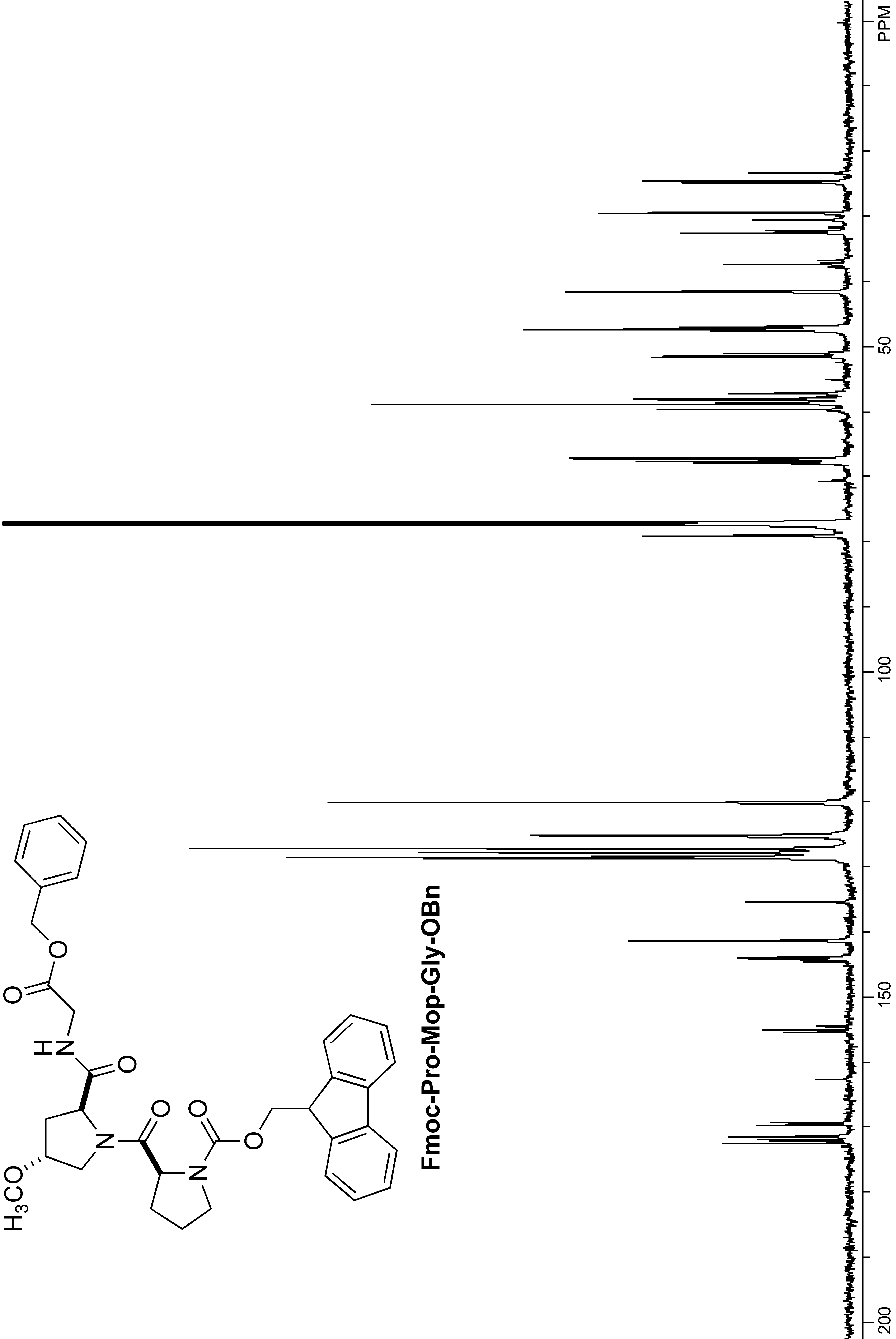


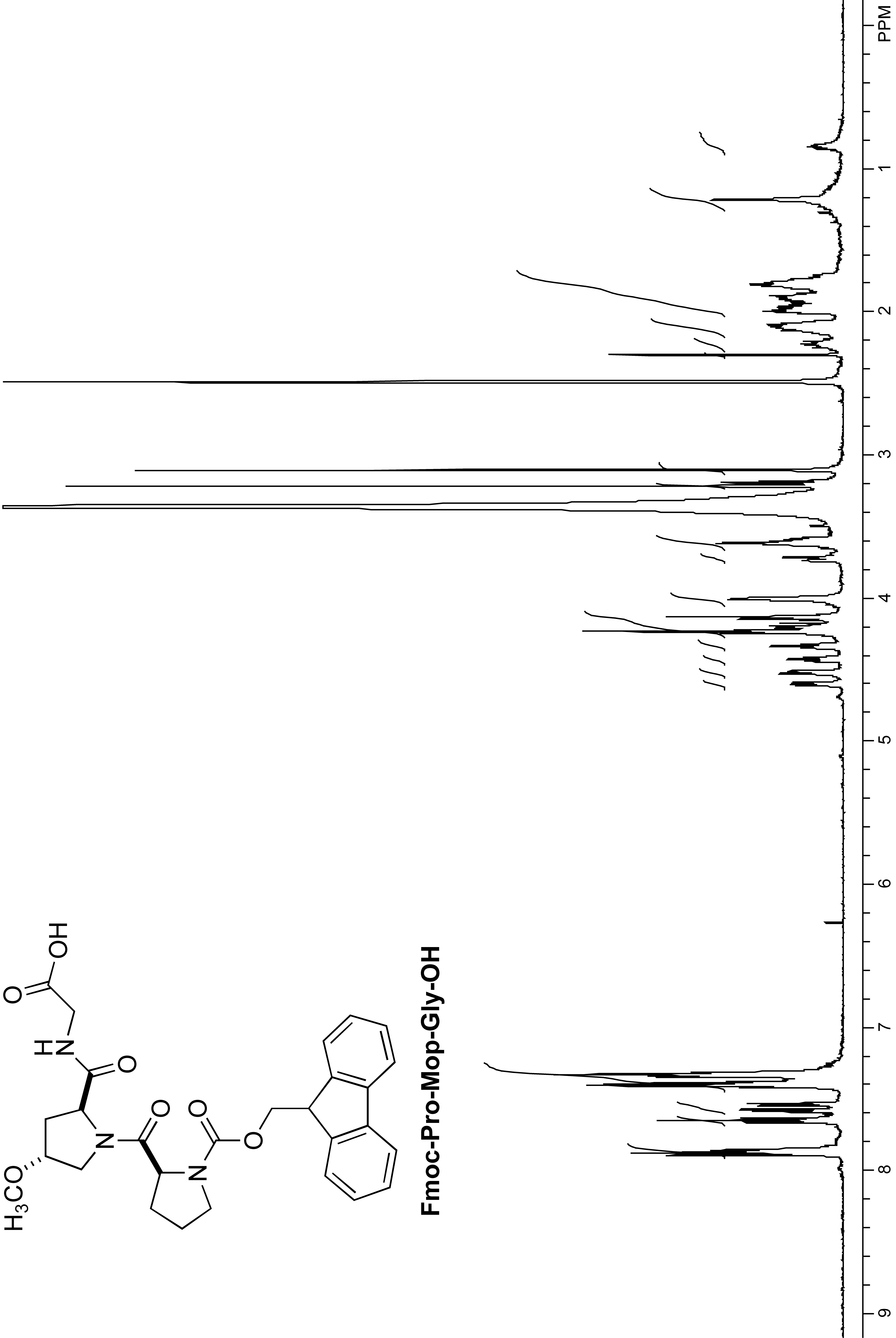


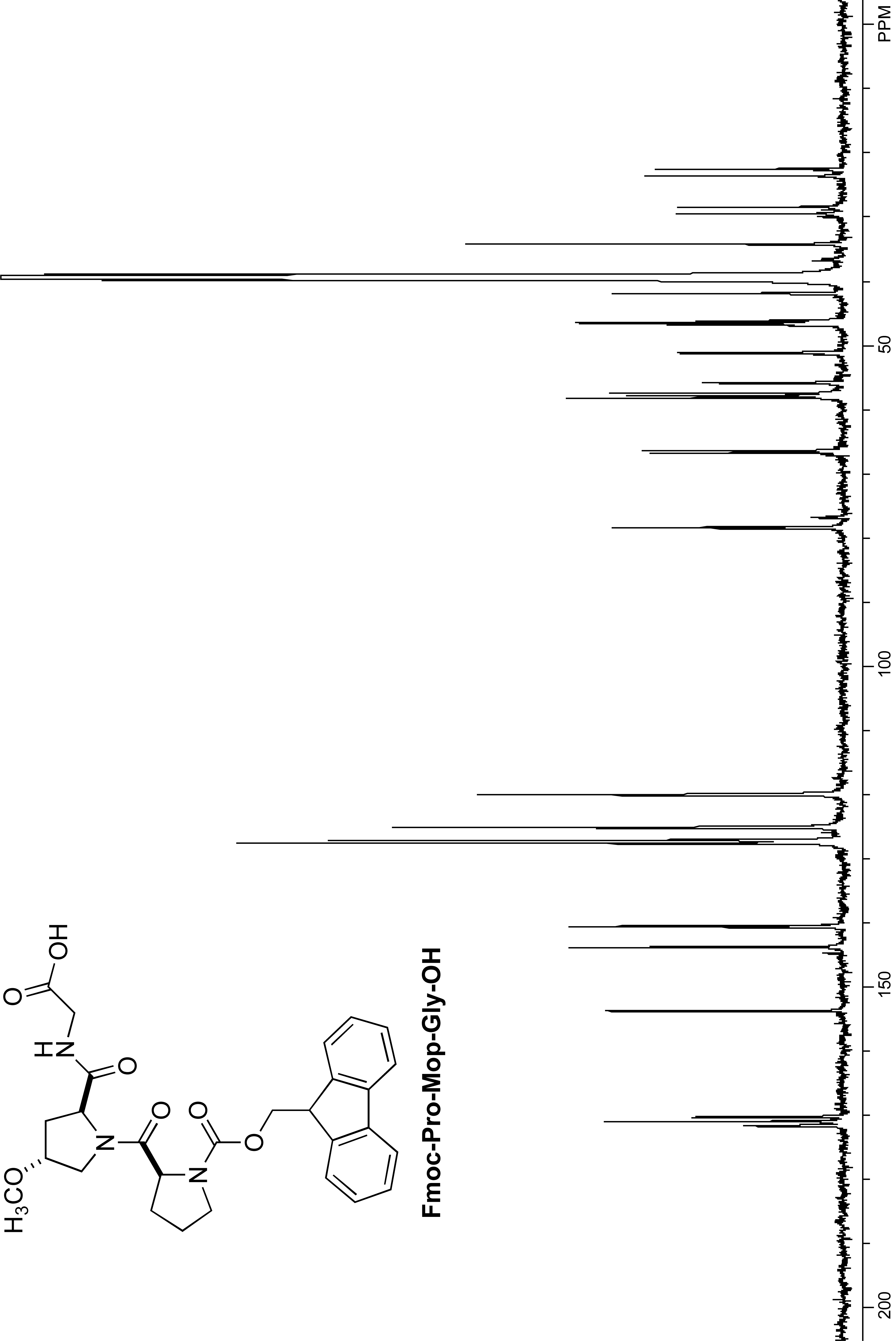


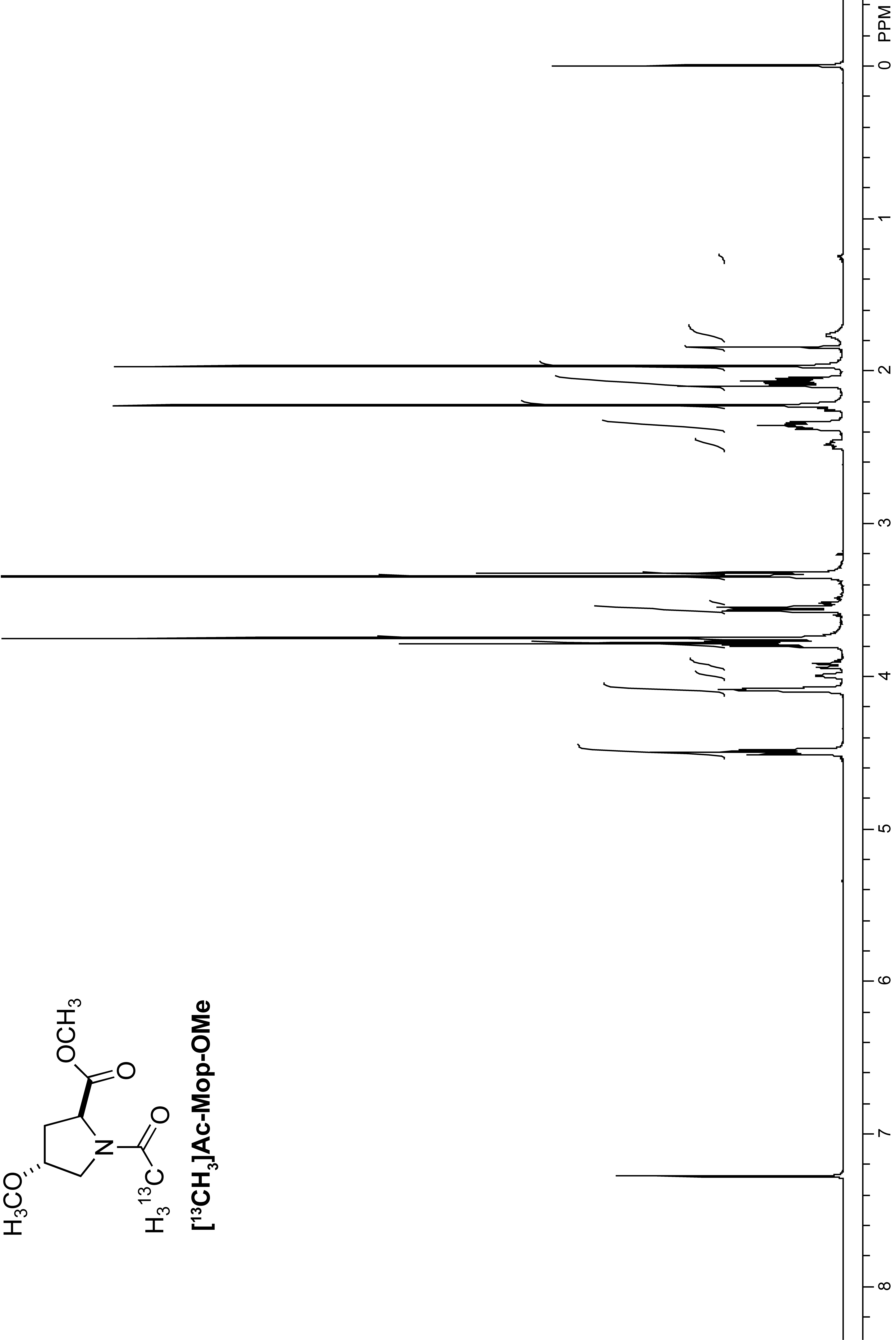


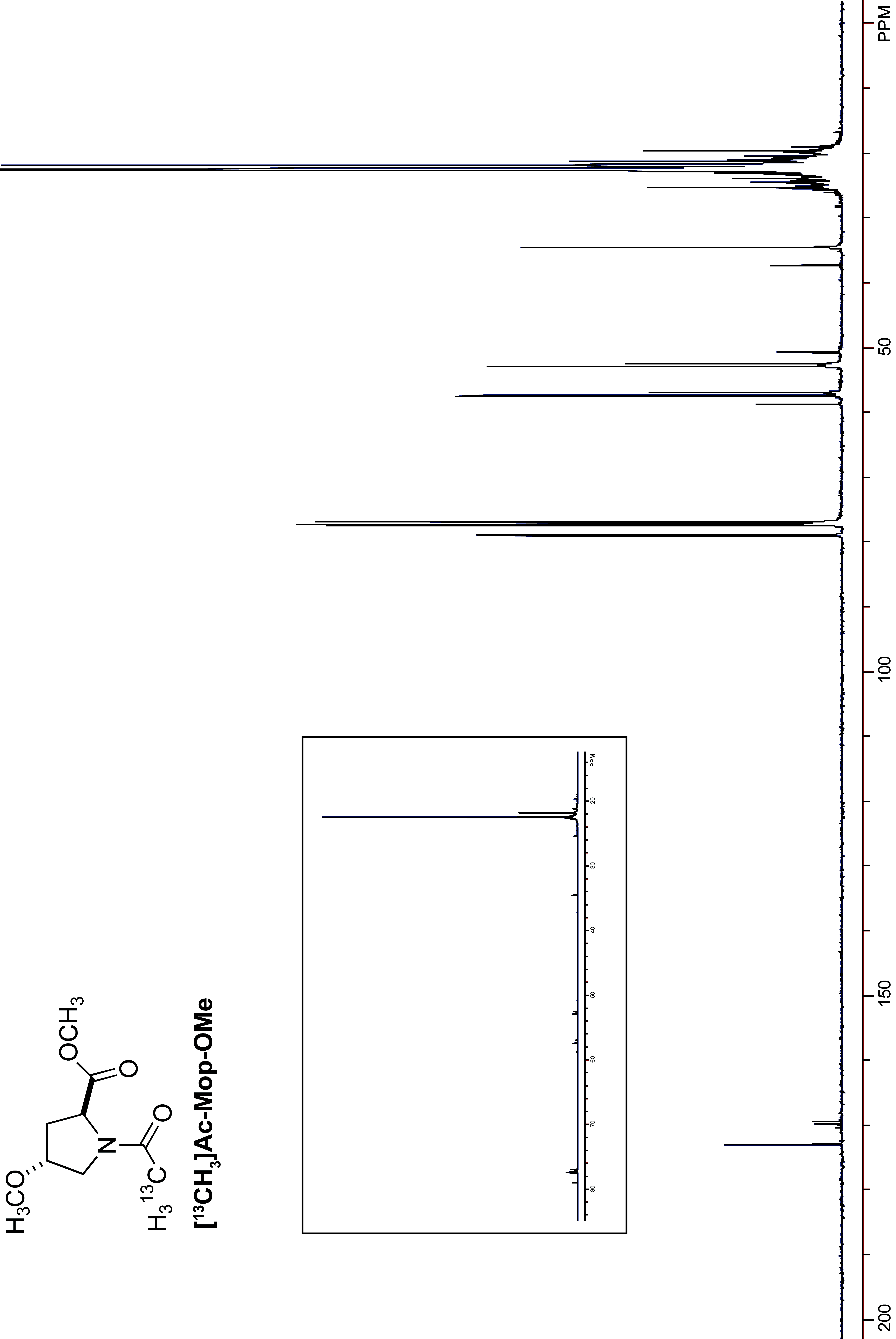

Supplement: Supporting_Information [file NIHMS167427-supplement-Supporting_Information.doc]
